# miR-335 Acts as a Tumor Suppressor and Enhances Ionizing Radiation-Induced Tumor Regression by Targeting ROCK1

**DOI:** 10.3389/fonc.2020.00278

**Published:** 2020-03-11

**Authors:** Yanfeng Cheng, Peng Shen

**Affiliations:** Department of Dermatology, Shengjing Hospital of China Medical University, Shenyang, China; Department of Orthopedics, Shengjing Hospital, China Medical University, Shenyang, China

**Keywords:** melanoma, ionizing radiation, resistance, apoptosis, miR-335, ROCK1

## Abstract

Recent development of integrative therapy against melanoma combines surgery, radiotherapy, targeted therapy, and immunotherapy; however, the clinical outcomes of advanced stage and recurrent melanoma are poor. As a skin cancer, melanoma is generally resistant to radiotherapy. Hence, there is an urgent need for evaluation of the mechanisms of radioresistance. The present study identified miR-335 as one of the differential expression of miRNAs in recurrent melanoma biopsies post-radiotherapy. The expression of miR-335 declined in melanoma tissues compared to the adjacent tissues. Moreover, miR-335 expression correlated with advanced stages of melanoma negatively. Consistent with the prediction of STARBASE and miRDB database, miR-335 targeted ROCK1 via binding with 3′-UTR of ROCK1 directly, resulting in attenuation of proliferation, migration, and radioresistance of melanoma cells. The authors validated that overexpression of miR-335 enhanced X-ray-induced tumor regression by B16 mouse models. Briefly, the present findings gained insights into miR-335/ROCK1-mediated radiosensitivity and provided a promising therapeutic strategy for improving radiotherapy against melanoma.

## Introduction

Malignant melanoma is the third frequently found skin cancer and characterized by early metastasis and poor diagnosis. Melanoma contributes to the majority of skin cancer-related deaths (1). Although the incidence of melanoma accounts for 1–3% among that of all kinds of cancer, the number of new cases each year in China reached 20,000 (2). In particular, the survival rate of patients with advanced stage melanoma is inadequate. The existing standard therapy against melanoma is an integrative treatment that combined surgery, radiation, targeted therapy, and immunotherapy. Given the successful control of *in situ* melanoma by radiotherapy, further investigation of the general radioresistance will probably provide a rationale to develop new radiosensitizers and improve radiotherapy.

MicroRNAs (miRNAs) are small RNA molecules with a length of about 22 nucleotides. miRNAs cause degradation or translation inhibition by binding to the 3′-untranslated region (UTR) of the target mRNA and play a regulatory role at the post-transcriptional level. miRNAs mediate multiple cellular functions, such as proliferation, differentiation, cycle progress, and apoptosis. Notably, miRNAs participate in the regulation of cell response to radiation (3, 4).

Many studies have confirmed that miR-335 plays a vital role in a variety of tumors. miR-335 worked as a tumor suppressor in a variety of tumors by inducing cell cycle arrest, promoting cell apoptosis, and changing the epigenetics of cell genome (5, 6). The overexpression of miR-335 improved the sensitivity of tumor cells to chemotherapy, including paclitaxel, cisplatin, and adriamycin (7). miR-335 inhibited TGF-β1-induced epithelial–stromal transformation of non-small cell lung cancer (NSCLC) through Rho-associated kinase 1 (ROCK1) (8). miR-335 also inhibits the proliferation, migration, and invasion of colorectal cancer cells by repressing LDHB (9). In particular, miR-335 targeted poly [ADP-ribose] polymerase 1 (PARP-1) directly and deregulated NF-κB expression, leading to enhancement of radiosensitivity (10). Currently, the knowledge of miR-335 in melanoma progression is limited.

The present study revealed that miR-335 upregulated in melanoma and targeted ROCK1. Besides, we demonstrated that the miR-335/ROCK1 axis was involved in the regulation of radioresistance *in vitro* and *in vivo*. Our findings illustrated that the mechanism underlying miR-335 worked as a radiosensitizer and provided insights for improving radiotherapy against melanoma.

## Materials and Methods

### Clinical Samples

We collected melanoma patients who came to Shengjing Hospital of China Medical University from May 2015 to June 2017. Among these patients, we selected 30 melanoma patients confirmed by three pathologists. The main criteria were (1) no other tumors, (2) no family history of malignant tumors, and (3) complete clinicopathological and follow-up information. After obtaining the informed consent, the fresh melanoma tissue and adjacent normal tissue samples within 5 cm from the tumor tissue (no tumor cells were confirmed by quick frozen section) were collected during the surgical removal process. Then, the removed samples were immediately washed with appropriate amount of cold saline to reduce blood pollution and stored in liquid nitrogen. The whole operation procedure was approved by the Medical Ethics Committee of Shengjing Hospital of China Medical University.

### Cell Culture

Melanoma cell lines A375, COLO829, HMCB PMWK, and B16 were obtained from China Center for Type Culture Collection (Wuhan, China). Cells were cultured in Dulbecco's modified Eagle's medium (DMEM, GIBCO BRL, Grand Island, NY, USA) supplemented with 10% fetal bovine serum (GIBCO BRL, Grand Island, NY, USA) in a humidified incubator containing 5% CO_2_ and split at 37°C. The cells in the irradiation group were irradiated with 4 Gy X-ray, and the cells in the control group were irradiated with 4 Gy X-rays.

### Plasmid Construction, RNA Interference, and Cell Transfection

miR-335 mimic, the scrambled control miR-control, miR-335 inhibitor, the negative control anti-miR-control, siRNA targeting ROCK1, and siRNA-negative control, as well as the recombinant lentiviruses encoding ROCK1 or empty vector control, were purchased from RiboBio (Guangzhou, China). To construct the luciferase-expressing vector plasmid, the target genes were amplified by PCR and cloned into the luciferase report vectors (Ambion, Foster City, CA, USA) to generate the wild-type reporter or mutant-type reporter. Analogously, the above oligonucleotides or plasmids were transfected into cells utilizing Lipofectamine 2000 reagent (Thermo Fisher Scientific, Waltham, MA, USA) referring to the manufacturer's instructions.

### RNA Extraction and Real-Time Polymerase-Chain Reaction (RT-PCR)

Total RNA was isolated from tissues or cells using TRIzol reagent (Invitrogen, Carlsbad, CA, USA) according to the instructions. RNA was quantified on NanoDrop ND-1000 (Applied Biosystems, Foster City, CA, USA) and synthesized complementary DNA using the Prime Script RT Reagent kit (Takara, Dalian, China) and the microRNA Reverse Transcription Kit (Thermo Fisher Scientific). RT-qPCR was carried out using the SYBR-Green PCR kit (Takara) on ABI 7500 HT system (Applied Biosystems). The fold changes of miRNA/mRNA levels were calculated by 2^−ΔΔCt^ method as previously described (11). Relative expression was normalized to glyceraldehyde-3-phosphate dehydrogenase (GAPDH) or endogenous small nuclear RNA U6. All primers for detecting gene expression are listed in Table 1.

**Table 1:** The sequences of the specific primers.

**Gene**	**Forward**	**Reverse**
miR-335	TCAAGAGCAATAACGAAAAATGT	GCGAGCACAGAATTAATACGAC
U6	CTCGCTTCGGCAGCACA	AACGCTTCACGAATTTGCGT
ROCK1	TCCTGCCAATTGTGATGCCT	GGGGAAGCACGAACAAAACC
CCND1	TCTACACCGACAACTCCATCCG	TCTGGCATTTTGGAGAGGAAGTG
CASP3	GGAAGCGAATCAATGGACTCTGG	GCATCGACATCTGTACCAGACC
GAPDH	TCROCKCAROCKCTTCCAGG	GATGACCCTTTTGGCTCCC

### miRNA Microarray Analysis

To evaluate the differentially expressed miRNAs in melanoma and adjacent tissues, we performed miRNA microarray analysis using a human miRNA microarray platform (miScript miRNA PCR Arrays, Cat No. 331221). PCR assays were performed using the Custom RT2 PCR Arrays (QIAGEN, Hilden, Germany) following the manufacturer's instructions. Reverse transcription was performed from 500 ng total RNA using the RT2 First Strand Kit (QIAGEN, Hilden, Germany). The data have been curated by the Gene Expression Omnibus database (accession number GSE143777).

### Luciferase Reporter Assay

Online database starBase v2.0 was utilized to predict potential binding sequences of miR-335. A375 cells were co-transfected with miR-335 mimic or the control plus 500 nM of ROCK1-WT/ROCK1-MUT using Lipofectamine 2000 according to the manufacturer's instructions. Luciferase activities were detected at 24 h after transfection using Dual-Luciferase Reporter Assay System (Thermo Fisher Scientific). The results were expressed as relative luciferase activity, and luciferase of Renilla was used as internal control.

### 3-(4,5-Dimethylthiazol-2-yl)-2,5-Diphenyltetrazolium Bromide (MTT) Assay

Exponentially growing cells were seeded onto 96-well plates in DMEM. Subsequently, 20 μl of MTT (Beyotime Biotechnology, China; 5 mg/ml) was added to each well and cultured for an additional 4 h. Dimethyl sulfoxide (DMSO) was used to dissolve the generated formazan crystals after discarding the supernatant. In general, cell viability was determined by measuring absorbance at 490 nm wavelength on a microplate reader (Applied Biosystems) at 0, 12, 24, 36, and 48 h post-transfection, respectively.

### Cell Cycle Analysis

Cells (1 × 10^5^) were exposed to X-rays immediately post-transfection with miRNA control or miR-335, followed by cell cycle distribution analysis 48 h later. Cell cycle status was determined with the Propidium Iodide Flow Cytometry Kit (Abcam, Cambridge, UK), followed by the manufacturer's protocol. At least three independent experiments were conducted.

### Apoptosis Assay

Cells (1 × 10^5^) were exposed to X-rays immediately post-transfection with miRNA control or miR-335, followed by apoptosis analysis 48 h later. Cell apoptosis was accessed with the annexin V-fluorescein isothiocyanate (FITC) detection kit (Abcam, Cambridge, UK) according to the manufacturer's protocol. Five microliters of Annexin V-FITC and 5 μl of propidium iodide were used to stain cells for 30 min at room temperature in the dark. Flow cytometry was performed to analyze apoptosis with the BD FACSCalibur Flow Cytometry System (BD Biosciences, Franklin Lakes, NJ, USA). 1 × 10^5^ gated events were gained from each sample, and at least three independent experiments were carried out.

### Cell Migration Assay

Melanoma cells (2 × 10^5^) were seeded into the transwell upper chamber wells of a 24-well plate with 8-μm poly(ethylene terephthalate) membranes (Millipore Corp., Billerica, MA, USA) in medium, while lower wells contained a complete medium with 10% fetal bovine serum. After incubation for 48 h, cotton swabs were used to remove non-migrating cells. The migrated cells were fixed with 20% methanol and stained with 0.1% crystal violet (Beyotime Biotechnology, China). Cell migration assay was analyzed by counting cells in five random fields from each of the migration chambers under a microscope (Applied Biosystems).

### Western Blot Assay

Cells were harvested with RIPA buffer (Beyotime Biotechnology, China). Then, the protein concentration was examined using BCA Protein Assay Kit (Beyotime Biotechnology, China). Equal amounts of protein samples were loaded on sodium dodecyl sulfate polyacrylamide gel electrophoresis (SDS-PAGE) and transferred onto polyvinylidene fluoride (PVDF) membranes. Subsequently, membranes were blocked with 5% skimmed milk for 2 h at room temperature and probed with primary antibodies ROCK1 (sc-365628), Cyclin D1 (sc-8396), Caspase3 (sc-7272), phosphor-Cofilin (sc-271921), Cofilin (sc-376476), and GAPDH (sc-47724) at 4°C overnight. All the primary antibodies were purchased from Santa Cruz Biotechnology (Dallas, TX, USA) and diluted at 1:1000. GAPDH was used for normalization. After extensive washing, the membranes were incubated with a goat anti-rabbit horseradish peroxidase-conjugated secondary antibody for 1 h. Finally, protein bands were visualized and analyzed with an enhanced chemiluminescent method.

### Generation of Mouse Models of Melanoma Transplantation

Six-week-old female BAB/c nude mice were disinfected, cell suspension was absorbed and injected subcutaneously at the back of both sides of the mice with 0.2 ml/side of cell suspension, and 3 × 10^5^ B16 cells were injected into each side according to the standard procedure (12). After inoculation, the tumor body was observed every day. After 8 days, 18 mice that harbored 100 mm^3^ tumors were divided into three groups (6 per group), followed by different group interventions. Sixteen mice were randomly divided into two groups: the irradiation group and the treatment group (*n* = 8). The next day, the mice received irradiation with 4 Gy X-rays. After fixing the mice, the mice were raised to the top and the lead plate covered the whole body. The holes were drilled corresponding to the tumor body to conduct the local irradiation of the tumor body. Each use required the recalibration of the dose rate. The samples were irradiated vertically from top to bottom. The irradiation time was adjusted according to the actual dose rate. Each tumor in the treatment group was injected with 10 μg of miR-335 every other day according to the previous protocols (13), and each tumor in the control group was injected with 10 μg of control.

Measurement of tumor volume: after fixation, the tumor was exposed, and the longest diameter (A) and the shortest diameter (B) of the tumor were measured. According to the calculation formula of tumor volume: *V* = *A* × *B*^2^/2, the total volume was calculated. At the same time, the tumor volume growth curve was recorded after recording daily.

### Immunohistochemical Staining

The tumor was fixed overnight in 4% paraformaldehyde solution. After embedding, the samples were cut into 4 μm thin slices, followed by standard immunohistochemical staining procedure with the primary antibody against ROCK1 at a dilution of 1:50. Finally, each slide was photographed and recorded.

### Statistical Analysis

All quantitative data were expressed as mean ± standard deviation. The difference between two groups was estimated by the Student's *t*-test and Pearson correlation analysis. GraphPad Prism 7 (GraphPad Inc., La Jolla, CA, USA) was performed to assess *P*-value among three or more groups with one-way analysis of variance. Statistically significant was defined at a *P* < 0.05.

## Results

### miR-335 Expression Decreases in Melanoma and Targets ROCK1

**Figure 1:**
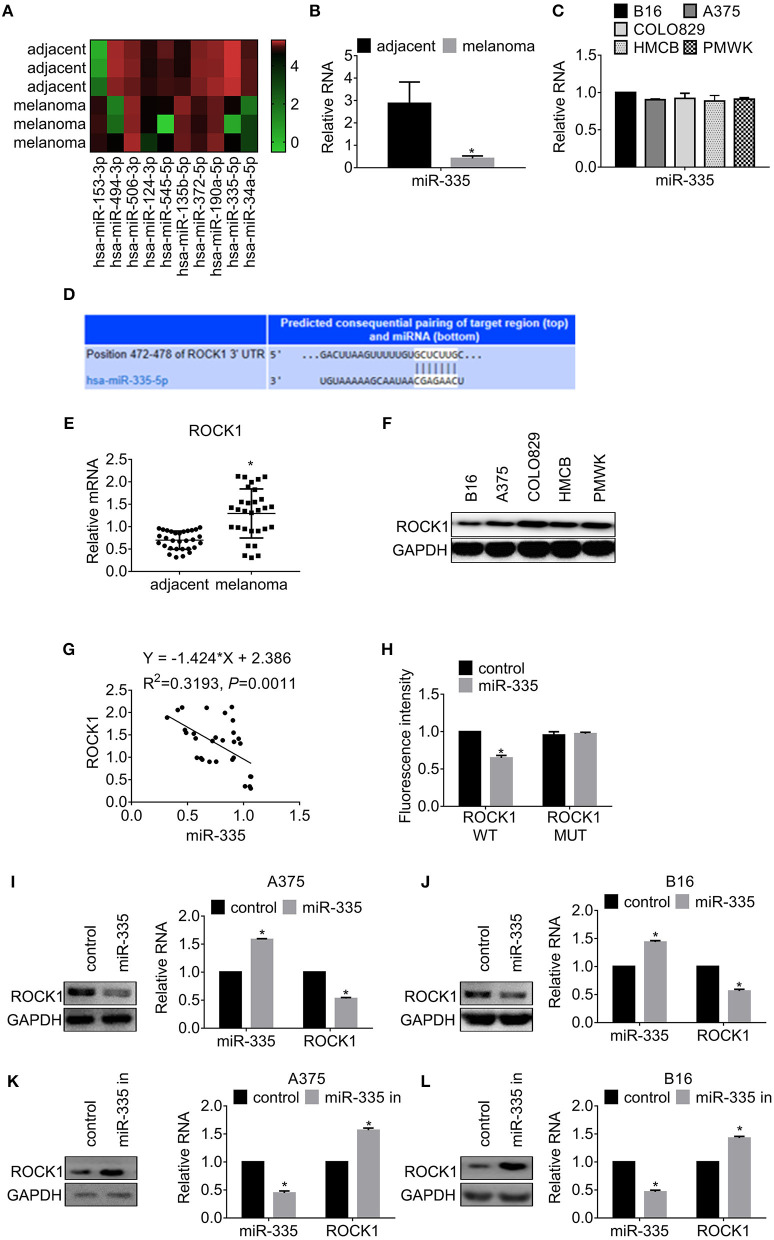
miR-335 expression declined in melanoma and targeted ROCK1. **(A)** MicroRNA array analysis was used to investigate the differential expression of miRNAs in melanoma and adjacent tissues. **(B)** The expression of miR-335 in 30 pairs of melanoma samples. *P < 0.05 vs. adjacent tissues. **(C)** The expression of miR-335 in B16, A375, COLO829, HMCB, and PMWK cells. **(D)** Putative complementary sites of miR-335 on 3′ UTR of ROCK1. **(E)** The expression of ROCK1 in 30 pairs of melanoma tissues and adjacent tissues. *P < 0.05 vs. adjacent tissues. **(F)** ROCK1 expression in B16, A375, COLO829, HMCB, and PMWK cells. **(G)** Correlation between ROCK1 and miR-335 expression in melanoma tissues. **(H)** The dual-luciferase activity of ROCK1 WT and ROCK1 MUT along with miR-335. ROCK1 WT, wild-type 3′-UTR of ROCK1; ROCK1 MUT, mutated 3′-UTR of ROCK1. *P < 0.05 vs. WT. **(I,J)** The ROCK1 expression in cells upregulating miR-335 was measured by Western blot or qRT-PCR. *P < 0.05 vs. control. **(K,L)** The ROCK1 expression in cells attenuating miR-335 was measured by Western blot or qRT-PCR. *P < 0.05 vs. control. Data gained in three independent experiments. The results are presented as the mean ± standard deviation.

According to the analysis of data obtained from miRNA microarray, we found several differential expressed miRNAs in melanoma compared to the corresponding adjacent tissues. miR-335 was the most significantly decreased among the miRNA (Figure 1A). We further examined miR-335 expression in 30 paired melanoma samples by real-time PCR. The data showed that miR-335 remarkably declined in melanoma tissues in comparison with the adjacent tissues (Figure 1B). We revealed that miR-335 expression was related to invasive depth, lymph node metastasis, and advanced stages of melanoma by correlation analysis (Table 2). Inversely, the miR-335 expression showed no significant connection with the gender of the ages of the patients with melanoma. We measured the miR-335 expression in murine tumor cell line B16 and human melanoma cell lines, including A375, COLO829, Human Melanoma Cell Bowes (HMCB), and PMWK. The results in Figure 1C demonstrated that the miR-335 generally expressed in immortalized melanoma cell lines.

**Table 2:** The relationship between miR-335 expression and melanoma features.

**Parameters**	**Description**	**No. of patients**	**miR-335 expression**		**χ^**2**^**	***P-*value**
			**Low**	**High**		
Gender	Male	19	13	6	0.578	0.447
	Female	11	6	5		
Age (years)	<50	8	7	1	2.744	0.098
	≥50	22	12	10		
Depth of invasion (pT)	T1, T2	7	2	5	4.751	0.029^*^
	T3, T4	23	17	6		
Lymph node metastasis (pN)	No	13	5	8	6.111	0.013^*^
	Yes	17	14	3		
TNM stage	I	7	2	5	7.364	0.025^*^
	II	6	4	2		
	III	17	16	1		

* *P < 0.05 indicated significant differences*.

Next, we employed StarBase and MIRDB to identify potential targets of miR-335. Based on the prediction, we selected ROCK1 for the subsequent investigation. The schematic of binding sites between ROCK1 and miR-335 is presented in Figure 1D. We found that the expression of ROCK1 in melanoma tissues increased significantly compared to that in adjacent tissues (Figure 1E). Besides, ROCK1 expressed widely in the melanoma cell lines (Figure 1F). The negative correlation between the expression of miR-335 and ROCK1 was estimated by Spearman's correlation analysis (Figure 1G). We, therefore, conducted the dual-luciferase assay to access the effect of miR-335 on the transcription of ROCK1. Cells were transfected with wild-type 3′-UTR of ROCK1 (ROCK1 WT) or mutated 3′-UTR of ROCK1 (ROCK1 MUT) along with miR-335, followed by a dual-luciferase assay. The results in Figure 1H exhibited that the relative luciferase activity of wild-type 3′-UTR of ROCK1 reduced 50% while that of mutated stayed firmly.

Additionally, we further investigated the expression of ROCK1 in cells expressing miR-335 mimic or inhibitor. As shown in Figures 1I,J, overexpression of miR-335 inhibited ROCK1 expression in translational and transcriptional levels compared to the control group. Conversely, the introduction of miR-335 inhibitor promoted ROCK1 expression in protein and mRNA levels (Figures 1K,L).

### miR-335 Suppresses Proliferation and Migration, While Enhancing Cell Cycle Arrest and Apoptosis by Repressing ROCK1

**Figure 2:**
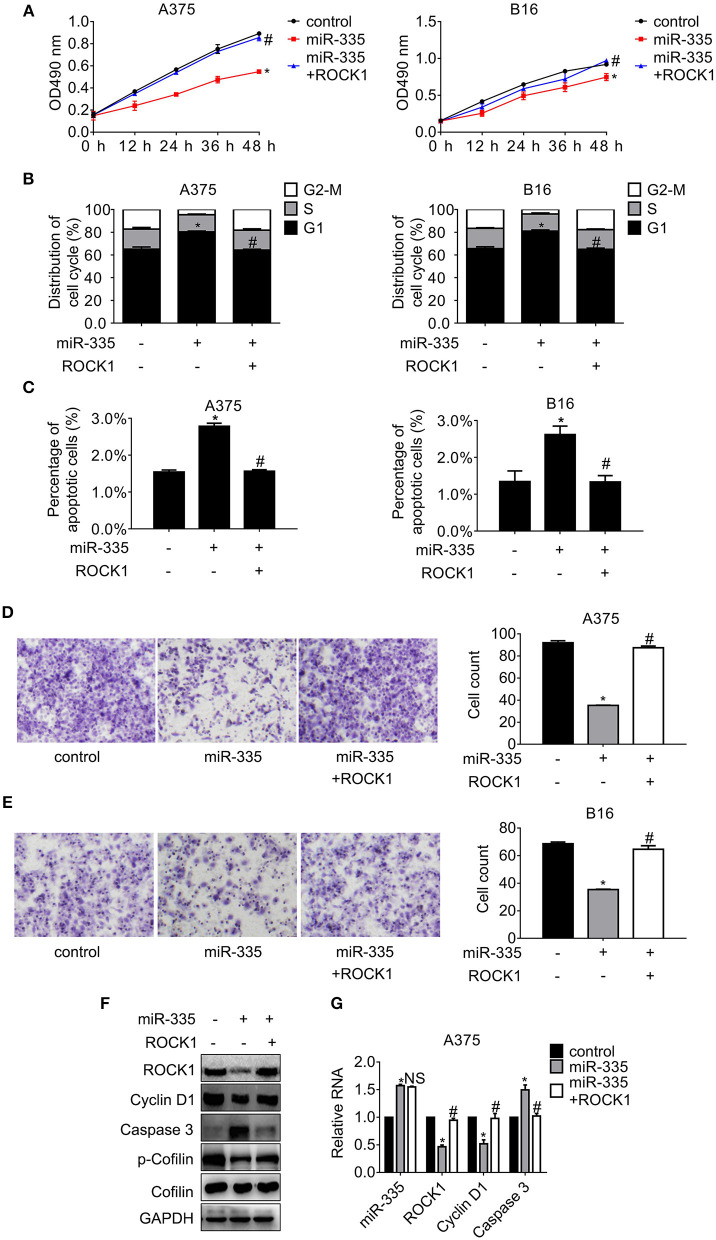
Overexpression of miR-335 suppressed proliferation and migration, while promoting G1 phase arrest and apoptosis by targeting ROCK1. **(A)** The cell proliferation of A375 and B16 cells expressing miR-335 or miR-335 plus ROCK1 at the indicated time points. **(B)** The cell cycle distribution of A375 and B16 cells expressing miR-335 or miR-335 plus ROCK1. **(C)** Apoptosis of A375 and B16 cells expressing miR-335 or miR-335 plus ROCK1. **(D,E)** Migration of A375 and B16 cells expressing miR-335 or miR-335 plus ROCK1. **(F,G)** The expression of the indicated genes in the cells expressing miR-335 inhibitor or the combination was determined by Western blot and qRT-PCR, respectively. *P < 0.05 vs. control, ^#^*P* < 0.05 vs. miR-335. Data gained in three independent experiments. The results are presented as the mean ± standard deviation.

To further explore the effect of miR-335 on proliferation and migration of melanoma cells, a series of follow-up experiments were conducted. The data in Figure 2A indicated that ectopic expression of miR-335 inhibited cell proliferation, while ROCK1 restored the inhibition compared to the control group. Cell cycle distribution and apoptosis assay were analyzed by flow cytometry consequently. Figure 2B showed that miR-335 promoted the population of G1 phase evidently while the ROCK1 expression reversed the enhancement of G1 phase in comparison with the control. The results in Figure 2C proved that the percentage of apoptotic cells expressing miR-335 increased ~1-fold while that expressing miR-335 plus ROCK1 altered hardly vs. the control. Besides, cell migration significantly decreased in cells expressing miR-335, while it was recovered by ROCK1 (Figures 2D,E).

Additionally, the changes of the pivotal regulators involved in ROCK1-mediated signaling pathways were examined by Western blot and RT-PCR, respectively (Figures 2F,G). The critical protein of G1-S phase transition, Cyclin D1, and the phosphorylation of the actin-modulating protein, Cofilin, dropped apparently, whereas the pro-apoptotic protein Caspase 3 raised accompanied by the deregulation of ROCK1 by miR-335. On the contrary, overexpression of ROCK1 impaired the miR-335 suppression, leading to rehabilitation of Cyclin D1 and phosphor-Cofilin and the downregulation of Caspase 3. Identical results were observed in B16 cells (Supplementary Figure 1A).

### Attendance of miR-335 Triggers Cell Proliferation and Migration, While Inhibiting Cell Cycle Progress and Apoptosis by Recovering ROCK1

**Figure 3:**
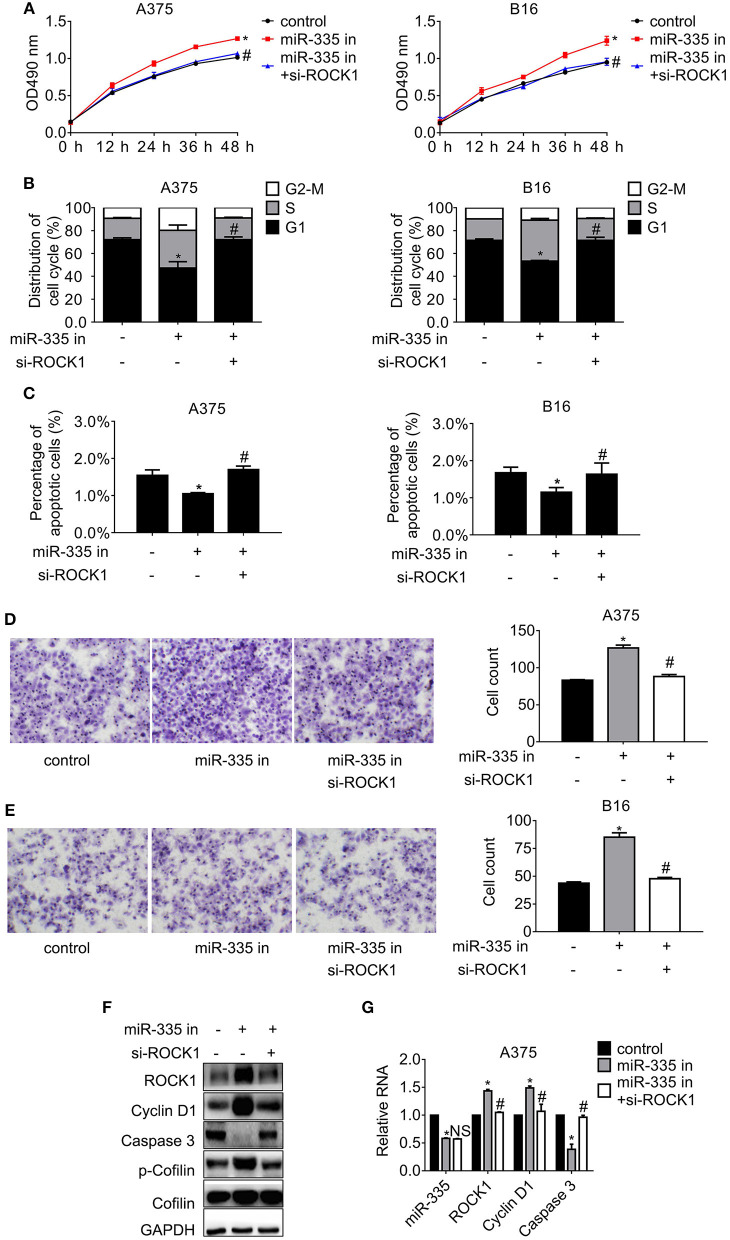
Inhibition of miR-335 enhanced proliferation and migration, while disturbing G1 phase arrest and apoptosis by recovering ROCK1. **(A)** The cell proliferation of A375 and B16 cells expressing miR-335 inhibitor or miR-335 inhibitor plus si-ROCK1 at the indicated time points. **(B)** The cell cycle distribution of A375 and B16 cells expressing miR-335 inhibitor or the combination. **(C)** Apoptosis of A375 and B16 cells expressing miR-335 inhibitor or the combination. **(D,E)** Migration of A375 and B16 cells expressing miR-335 or miR-335 plus ROCK1. **(F,G)** The expression of the indicated genes in the cells expressing miR-335 inhibitor or the combination was determined by Western blot and RT-PCR, separately. *P < 0.05 vs. control, ^#^*P* < 0.05 vs. miR-335 inhibitor. Data gained in three independent experiments. The results are presented as the mean ± standard deviation.

To illustrate the effect of silencing miR-335 on proliferation and migration of melanoma cells, experiments with miR-335 inhibitor or miR-335 inhibitor combined with si-ROCK1 were performed. The results in Figure 3A suggested that knockdown of miR-335 induced cell proliferation, while siRNA targeting ROCK1 hampered the proliferation compared to the control group. The efficacy of ROCK1 interference by siRNA was validated by immunoblot (Supplementary Figure 1B). Besides, Figure 3B showed that the miR-335 inhibitor reduced the population of G1 phase notably, while the obstacle of ROCK1 expression by siRNA confronted the reduction of cells in G1 phase compared to the control. The results in Figure 3C demonstrated that the percentage of apoptotic cells deregulating miR-335 fell ~30%, while that of the combination group changed little vs. the control group. Moreover, the numbers of migrated cells evidently increased in cells with downregulation of miR-335, while that of the combination group returned to similar accounts of the control group (Figures 3D,E).

We further detected the changes in the previously mentioned interesting genes involved in ROCK1-regulated cell cycle progression, apoptosis, and motility. Cyclin D1 and the phosphorylation of Cofilin escalated significantly, whereas Caspase 3 diminished, accompanied by the deregulation of miR-335. Impairment of ROCK1 antagonized the results of miR-335 suppression, oppositely leading to the reduction of Cyclin D1 and phosphor-Cofilin and the enhancement of Caspase 3 (Figures 3F,G). Likewise, the same alterations were gained in B16 cells (Supplementary Figure 1C).

### miR-335 Promotes Melanoma Cell Response to Irradiation

**Figure 4:**
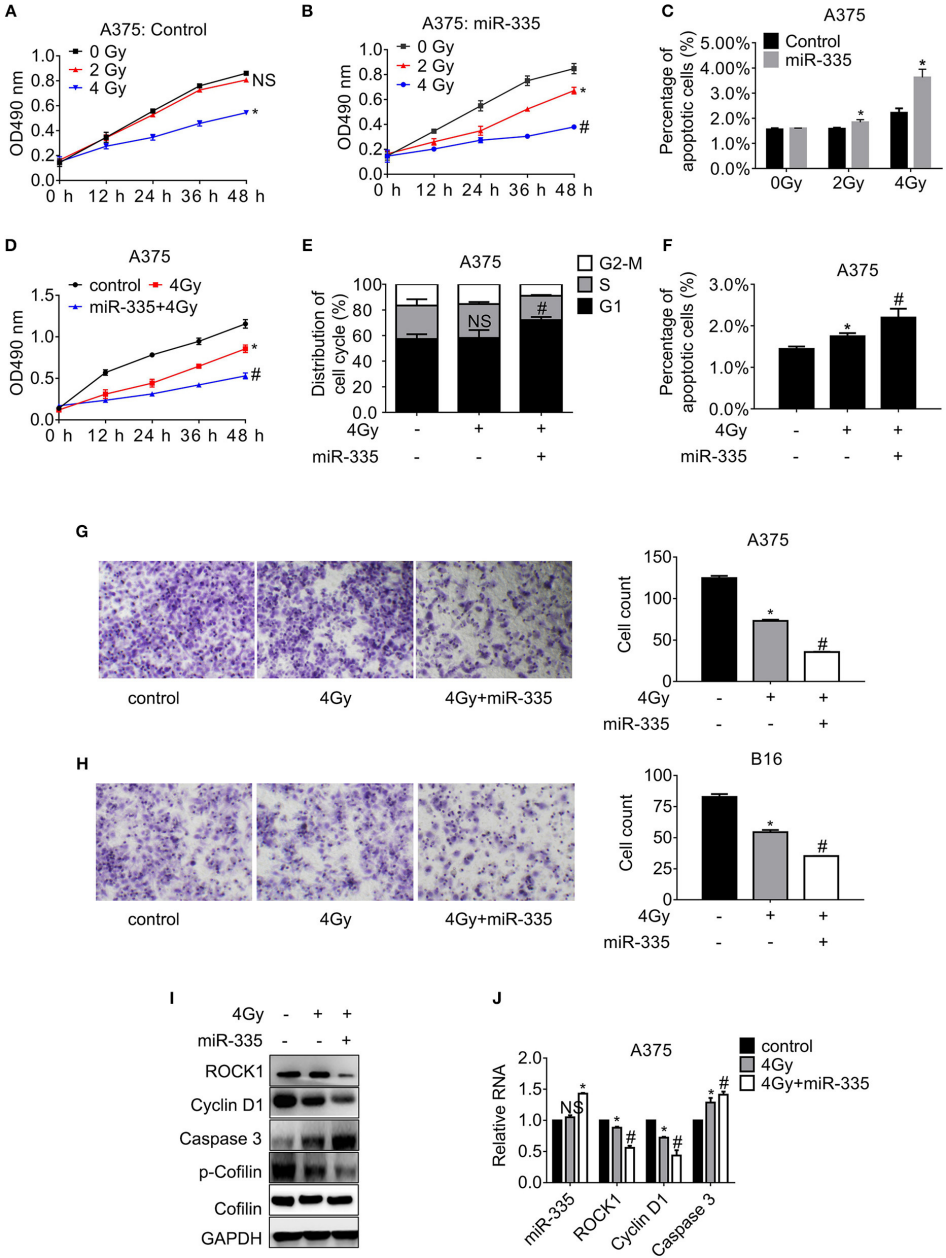
miR-335 promoted radiosensitivity of melanoma cells via ROCK1-mediated signaling pathways. **(A,B)** The proliferation of A375 cells expressing control or miR-335 post-irradiation or the combined treatment at the indicated time points. **(C)** The apoptosis of A375 cells expressing control or miR-335 post-irradiation or the combined treatment. **(D)** The proliferation of A375 cells post-4 Gy irradiation or the combined treatment. **(E)** The distribution of cell cycle of A375 cells post-4 Gy irradiation or the combined treatment. **(F)** The apoptosis of A375 cells post-4 Gy irradiation or the combined treatment. **(G,H)** Migration of A375 and B16 cells post-exposure to 4 Gy X-ray or the combined treatment. **(I,J)** The expression of the indicated genes in the cells received radiation or the combined treatment was accessed by Western blot and qRT-PCR, respectively. *P < 0.05 vs. control, ^#^*P* < 0.05 vs. 4 Gy group. Data gained in three independent experiments. The results are presented as the mean ± standard deviation.

To observe whether miR-335 associates the sensitivity of melanoma cells to irradiation, cells expressing miR-335 or control were exposed to X-ray, followed by proliferation and migration assay, as well as the flow cytometry analysis. As presented in Figure 4A, the proliferation of A375 cells expressing control declined significantly post-4 Gy irradiation while it altered little post-2 Gy irradiation. The proliferation of A375 cells expressing miR-335 dropped evidently post-2 Gy irradiation, indicating that miR-335 triggered the radiosensitivity of A375 cells (Figure 4B). The results in B16 cells were consistent with those in A375 cells (Supplementary Figures 1D,E). Besides, the apoptosis of cells expressing miR-335 increased to a greater extent compared to that expressing control post-irradiation (Figure 4C and Supplementary Figure 1F). The results were in line with the changes in cell proliferation.

According to the 4 Gy irradiation-induced apparent alteration of proliferation and apoptosis, the same dose was selected to treat cells in the subsequent experiments. The results in Figure 4D showed that miR-335 enhanced the 4 Gy inhibition on A375 cell proliferation significantly in comparison with the 4 Gy alone. Similar results were gained in B16 cells (Supplementary Figure 1G). Moreover, the cell cycle distribution and apoptosis of cells expressing control or miR-335 post-irradiation were accessed. The results in Figure 4E exhibited that 4 Gy X-ray had little effect on the cell cycle progress of A375 cells that expressed the scrambled control. In contrast, 4 Gy X-ray notably induced increment of G1 population in cells expressing miR-335. Likewise, the population of G1 cells in the miR-335 group markedly increased while that in the control group remained stable (Supplementary Figure 1H). Furthermore, Figure 4F indicated that the miR-335 enhanced the stimulation of apoptosis by irradiation in comparison with control. Identical results were seen in B16 cells (Supplementary Figure 1I). Apart from enhanced inhibition of cell proliferation, we found that 4 Gy X-ray retarded the migration of cells expressing miR-335 to a greater extent compared to that expressing control (Figures 4G,H).

We further confirmed that Cyclin D1 and the phosphor-Cofilin in A375 cells expressing miR-335 dropped to a more considerable extent compared to that in the control post-irradiation. Inversely, Caspase 3 in cells expressing miR-335 elevated to a higher degree in comparison with that expressing control after receiving irradiation (Figures 4I,J). The identical alterations were validated in B16 cells (Supplementary Figure 1J).

### miR-335 Elicits Radiosensitization in B16 Mouse Model

**Figure 5:**
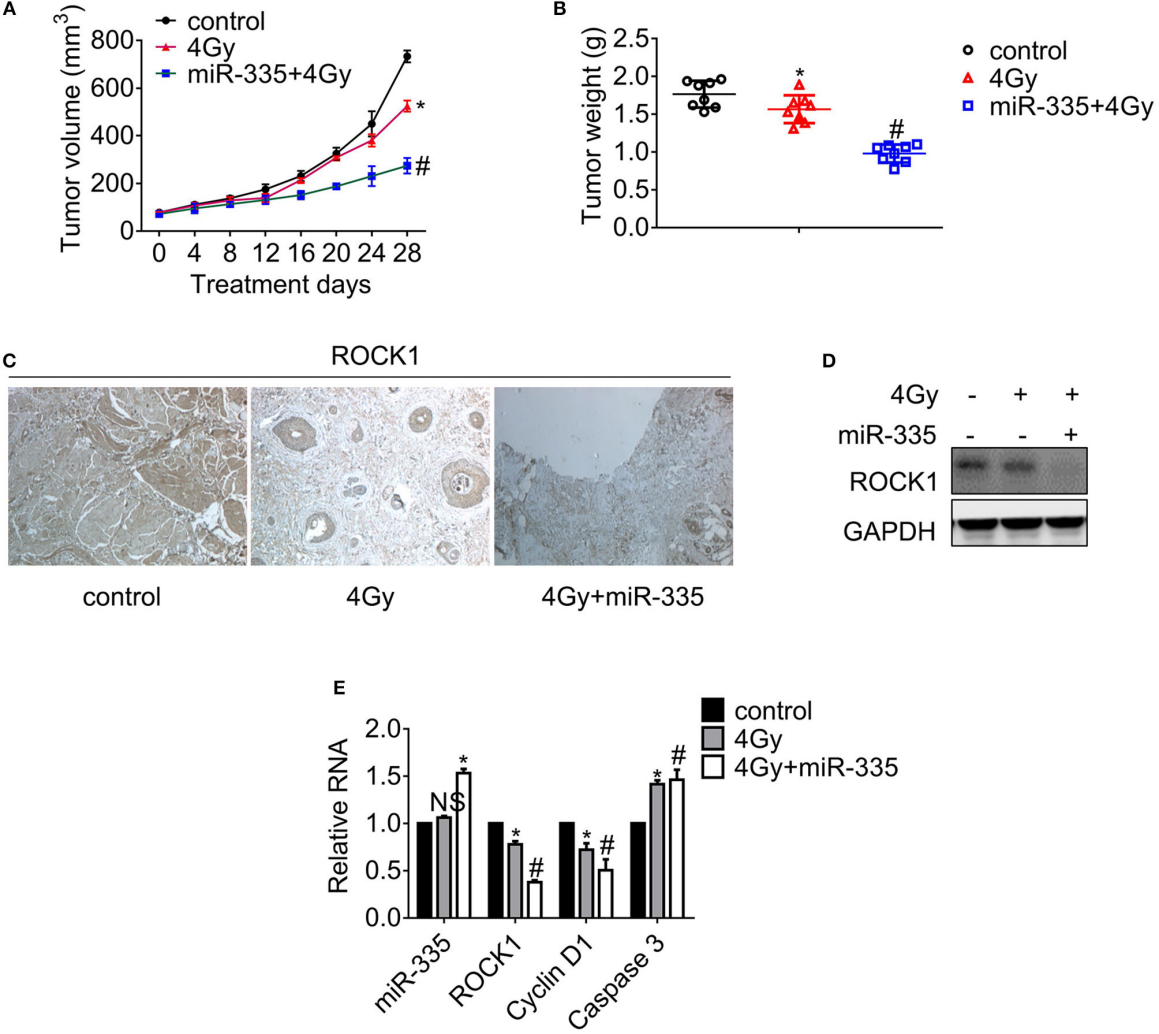
miR-335 promoted radiosensitivity of melanoma in A375 mouse models. Mice with 100–300 mm^3^ tumors were exposed to X-ray or not. **(A)** Tumor growth curve of melanoma in xenografts. **(B)** Tumor weight of melanoma in xenografts. **(C)** Detection of ROCK1 expression in tumor tissues by immunohistochemistry. **(D)** The ROCK1 expression in tumors was assessed by Western blot. **(E)** The expression of the indicated genes was analyzed by RT-PCR. *P < 0.05 vs. control, ^#^*P* < 0.05 vs. 4 Gy group. Data gained in three independent experiments. The results present as the mean ± standard deviation.

Palpable tumors appeared on day 8 upon subcutaneous injection. The tumor volumes were measured 4 days, and the tumors were weighed after the mice were humanely sacrificed. The tumor growth curves shown in Figure 5A suggested that 4 Gy X-ray impeded the tumor growth slightly while the combination of irradiation and the *in situ* injection of miR-335 daily obstructed the tumor growth significantly. In line with the results of tumor growth, the tumor weights of dual treatment decreased to the greatest extent while those that received irradiation dropped slightly (Figure 5B). Besides, the ROCK1 expression in each tumor was examined by immunohistochemistry staining (Figure 5C), immunoblot (Figure 5D), and RT-PCR analysis (Figure 5E), respectively. The results validated that the dual treatment suppressed ROCK1 expression to a larger extent compared to the single irradiation, as well as the decrease of Cyclin D1 and the boost of Caspase 3.

## Discussion

It is well-documented that miRNAs have promising potential in the development of novel therapeutic strategies against cancer (3, 4, 14). For example, miR-155 inhibited the proliferation, invasion, and migration of melanoma by targeting CBL (15). miR-542, as an essential factor in the regulation of epithelial–mesenchymal transformation, was significantly deregulated in patients with melanoma. miR-542 elevation inhibited the development of melanoma by inhibiting the epithelial–mesenchymal transformation (16). miR-26b deregulated in melanoma and targeted the TRAF5–MAPK signaling pathway, inhibiting the growth of melanoma cells (17). In this study, we found many miRNAs expressed abnormally in melanoma tissue by microarray analysis and selected miR-335 as the research object. miR-335 declined significantly in melanoma and directly targeted the widely expressed ROCK1 in melanoma. As a member of the Rho-related protein kinase (ROCK) family, ROCK1 plays an essential regulatory role in different types of cancer. ROCK1 mediated the occurrence and development of melanoma in various ways, and obstruction of ROCK1 inhibited the proliferation and migration of melanoma cells (18). This study indicated that overexpression of miR-335 suppressed the proliferation, migration, and radioresistance of melanoma cells. By contrast, restoration of ROCK1 overcame the inhibition by miR-335. Therefore, miR-335 disturbed proliferation and migration, while promoting radiosensitivity of melanoma by inhibiting ROCK1.

The miR-335/ROCK1 axis regulated multiple functions, including tumor progression (19–21), myocardial ischemia/reperfusion (22), and chondrogenesis (23). Lynch and colleagues firstly illustrated the negative feedback loop among miR-335/ROCK1-noncanonical TGF-β signaling and MYCN (24). miR-335 inhibited the invasiveness of neuroblastoma via the complex regulatory network. miR-335 repressed TGF-β-induced epithelial–mesenchymal transition and proliferation by targeting ROCK1 in NSCLC (8, 25). Recently, several studies revealed that long noncoding RNA DANCR and NEAT1 promoted ROCK1-mediated signaling pathways via decoying miR-335, leading to enhancement of cancer malignancy (26–29). Previous studies implied that the miR-335/ROCK1 axis played diverse roles in the regulation of tumor initiation, progression, and relapse. Currently, the validated connection between miR-335/ROCK1 and radioresistance broadened the miR-335/ROCK1-mediated networks in the development of refractory melanoma.

Mounting evidence has validated the regulatory mechanisms of miRNA-mediated radiosensitization in various cancers. For instance, the overexpression of miR-32-5p contributed to radioresistance by targeting Transducer of ERBB2, 1 in colorectal cancer (30). miR-1275 elevation diminished epithelial-to-mesenchymal transition-induced radioresistance through Wnt/β-catenin signaling in esophageal cancer (31). Impediment of miR-365 suppressed the radiosensitivity of NSCLC via promoting cell division cycle 25A (32). The present study demonstrated that the downregulation of miR-335 led to radioresistance via suppressing ROCK1 in melanoma, expanding the understanding of miRNA-mediated radioresistance in the generally radioresistant skin cancer. Apart from the current study, Park and colleagues illustrated that Lin28B/let-7 interaction played a pivotal role in cancer stem cell-associated X-ray resistance (33). He et al. demonstrated that miR-185 enhanced the ionizing radiation-triggered inhibition of proliferation and migration (34). The abovementioned evidence suggested that miRNA played fundamental roles in radiosensitivity of melanoma.

Some shortages need to compensate in the current study. First, the response of melanoma to protons and carbon ions varies. The bystander effect and the abscopal effect, as well as the efficacy, may change during treatment. Hence, the details of miR-335/ROCK1 signaling in different regimens should be explored. Second, multiple signaling pathways such as Rho/ROCK, Ras/MAPK, and PI3K/Akt signaling cascades converge toward ROCK1; the complex interactions need more investigation. Third, the possible effects of BRAF mutation on the miR-335/ROCK1 axis need further illumination due to the frequent BRAF V600E in melanoma.

In conclusion, the present study demonstrated that miR-335 decreased in melanoma and targeted ROCK1. Overexpression of miR-335 inhibited cell proliferation and migration, while promoting radiosensitivity of melanoma cells. Inversely, the deregulation of miR-335 triggered proliferation and migration by restoration of ROCK1. The current evidence provided promising therapeutic miRNAs as targets for improving radiotherapy against melanoma.

## Data Availability Statement

The datasets generated for this study can be found in the NCBI Gene Expression Omnibus (GSE143777).

## Ethics Statement

The studies involving human participants were reviewed and approved by the Ethics Committee of Shengjing Hospital of China Medical University. The patients/participants provided their written informed consent to participate in this study. The animal study was reviewed and approved by the Ethics Committee of Shengjing Hospital of China Medical University.

## Author Contributions

YC wrote the main manuscript. YC and PS performed the experiments, designed the research, performed data analysis, and contributed to manuscript revisions. All authors reviewed the manuscript, read, and approved the final manuscript.

### Conflict of Interest

The authors declare that the research was conducted in the absence of any commercial or financial relationships that could be construed as a potential conflict of interest.

## Supplementary Material

Supplementary Figure 1 **(A)** The expression of indicated genes in the cells expressing miR-335 or miR-335 combined with ROCK1 was analyzed by Western blot and RT-PCR, respectively. ^*^*P* < 0.05, vs. control; ^#^*P* < 0.05, vs. miR-335. **(B)** The ROCK1 expression in cells with siRNA 1# AND 2# targeting ROCK1. **(C)** The expression of indicated genes in the cells expressing miR-335 inhibitor or miR-335 inhibitor combined with si-ROCK1 was analyzed by Western blot and RT-PCR, respectively. ^*^*P* < 0.05, vs. control; ^#^*P* < 0.05, vs. miR-335 inhibitor. miR-335 in, miR-335 inhibitor. si-ROCK1, siRNA targeting ROCK1. **(D,E)** The proliferation of B16 cells expressing control or miR-335 post-irradiation or the combined treatment at the indicated time points. **(F)** The apoptosis of B16 cells expressing control or miR-335 post-irradiation or the combined treatment. **(G)** The proliferation of B16 cells post-4 Gy irradiation or combined treatment. **(H)** The distribution of cell cycle of B16 cells post-4 Gy irradiation or the combined treatment. **(I)** The apoptosis of A375 cells post-4 Gy irradiation or the combined treatment. **(J)** The expression of indicated genes in the cells exposed to 4 Gy X-ray or radiation plus miR-335 was analyzed by Western blot and RT-PCR, respectively. ^*^*P* < 0.05, vs. control; ^#^*P* < 0.05, vs. 4 Gy. Data gained in three independent experiments. The results are presented as the mean ± standard deviation.
