# Corrigendum: miR-335 Acts as a Tumor Suppressor and Enhances Ionizing Radiation-Induced Tumor Regression by Targeting ROCK1

**DOI:** 10.3389/fonc.2021.650811

**Published:** 2021-02-04

**Authors:** Yanfeng Cheng, Peng Shen

**Affiliations:** ^1^ Department of Dermatology, Shengjing Hospital of China Medical University, Shenyang, China; ^2^ Department of Orthopedics, Shengjing Hospital, China Medical University, Shenyang, China

**Keywords:** melanoma, ionizing radiation, resistance, apoptosis, miR-335, ROCK1

In the published article, there was an error regarding the affiliations for Peng Shen. Instead of the affiliation “Department of Dermatology, Shengjing Hospital of China Medical University, Shenyang, China”, they should have the affiliation “Department of Orthopedics, Shengjing Hospital, China Medical University, Shenyang, China”.

The authors apologize for this error and state that this does not change the scientific conclusions of the article in any way. The original article has been updated.

