# Supplementary figures and images for: miR-335 Acts as a Tumor Suppressor and Enhances Ionizing Radiation-Induced Tumor Regression by Targeting ROCK1
Source: Front Oncol. 2020 Mar 11;10:278. doi: 10.3389/fonc.2020.00278 (PMC7078682; doi:10.3389/fonc.2020.00278)

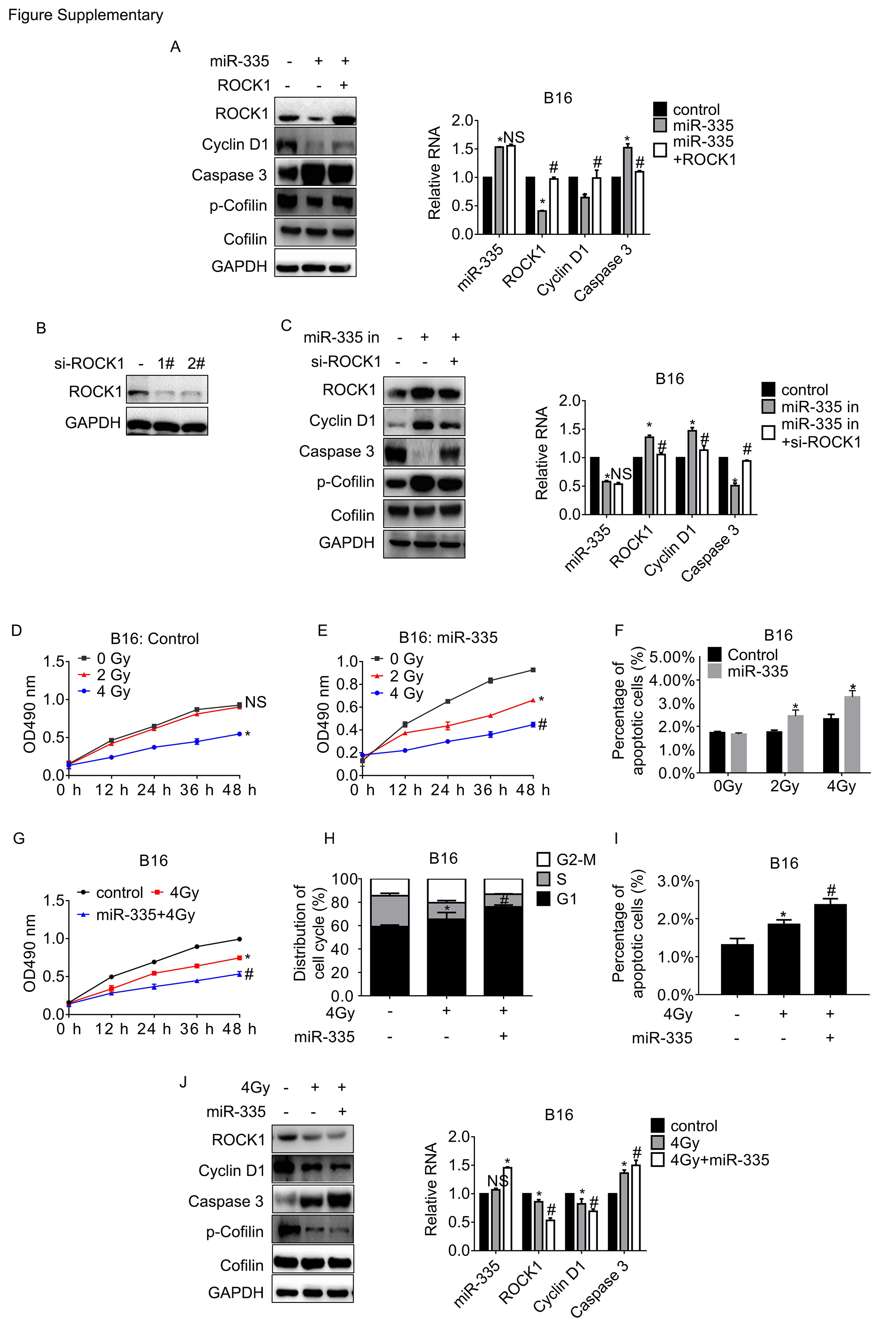

Supplement: Supplementary Figure 1 — (A) The expression of indicated genes in the cells expressing miR-335 or miR-335 combined with ROCK1 was analyzed by Western blot and RT-PCR, respectively. *P < 0.05, vs. control; #P < 0.05, vs. miR-335. (B) The ROCK1 expression in cells with siRNA 1# AND 2# targeting ROCK1. (C) The expression of indicated genes in the cells expressing miR-335 inhibitor or miR-335 inhibitor combined with si-ROCK1 was analyzed by Western blot and RT-PCR, respectively. *P < 0.05, vs. control; #P < 0.05, vs. miR-335 inhibitor. miR-335 in, miR-335 inhibitor. si-ROCK1, siRNA targeting ROCK1. (D,E) The proliferation of B16 cells expressing control or miR-335 post-irradiation or the combined treatment at the indicated time points. (F) The apoptosis of B16 cells expressing control or miR-335 post-irradiation or the combined treatment. (G) The proliferation of B16 cells post-4 Gy irradiation or combined treatment. (H) The distribution of cell cycle of B16 cells post-4 Gy irradiation or the combined treatment. (I) The apoptosis of A375 cells post-4 Gy irradiation or the combined treatment. (J) The expression of indicated genes in the cells exposed to 4 Gy X-ray or radiation plus miR-335 was analyzed by Western blot and RT-PCR, respectively. *P < 0.05, vs. control; #P < 0.05, vs. 4 Gy. Data gained in three independent experiments. The results are presented as the mean ± standard deviation. [file Image_1.jpeg]
